# Terahertz's silent revolution in physics, engineering, and life science: Beyond the spectrum

**DOI:** 10.1016/j.fmre.2025.05.004

**Published:** 2025-05-11

**Authors:** Yuankun Sun, Shaomeng Wang, Yubin Gong

**Affiliations:** a School of Electronic Science and Engineering, University of Electronic Science and Technology of China, Chengdu 611731, China; b National Key Laboratory of Science and Technology on Vacuum Electronics, University of Electronic Science and Technology of China, Chengdu 611731, China; c Terahertz Radiation and Application Key Laboratory of Sichuan Province, University of Electronic Science and Technology of China, Chengdu 611731, China

**Keywords:** Terahertz, Coherent detection, Terahertz channels, Terahertz metamaterial biosensors, Neuron modulation

## Abstract

Terahertz technology is revolutionizing photonics, biomedicine, and communications by merging non-ionizing radiation with molecular sensitivity and material penetration. Advances in metamaterials, adaptive antennas, and AI-driven systems address historical limitations in emission efficiency and atmospheric attenuation, enabling secure high-capacity networks and precision biomedical applications. Reconfigurable beamforming and hybrid channel models enhance wireless reliability, while ultra-sensitive biosensors and neuromodulation techniques pioneer non-invasive diagnostics and therapies for neurodegenerative and psychiatric disorders. Terahertz’s dual role in molecular sensing and neural modulation establishes closed-loop “detect-treat” paradigms, bridging material science and neuroscience. Challenges remain in optimizing clinical application and hybrid system scalability, yet its capacity to probe carrier dynamics, protein interactions, and neural circuits positions Terahertz as a universal platform for 6G networks, personalized medicine, and brain-machine interfaces. By unifying physics-aware engineering with biological insights, terahertz technology transcends traditional boundaries, offering transformative solutions for healthcare, secure connectivity, and industrial innovation.

## 1 Introduction

The terahertz (THz) band (0.1–10 THz), a unique electromagnetic window bridging photonics and electronics, has emerged as a transformative frontier in communication, biomedicine, and materials science. This spectral region combines non-ionizing radiation, material penetration capabilities, and strong resonance with molecular vibrational-rotational modes, offering unprecedented opportunities for non-destructive imaging, molecular fingerprint identification, and ultrafast wireless communication [1]. Unlike microwave and infrared technologies, THz waves penetrate non-polar materials while selectively exciting biomolecular interactions, enabling high-resolution sensing and diagnostics. Conventional techniques (e.g., optical spectroscopy or X-ray imaging) often face inherent constraints, such as limited penetration depth, ionizing radiation risks, or water interference. Such limitations highlight THz’s unique potential in bridging these gaps. However, the research of THz technology has long been hindered by challenges such as inefficient emission sources, low-sensitivity detectors, and complex propagation dynamics in atmospheric and biological environments. Recent breakthroughs in metamaterials, quantum cascade lasers, and artificial intelligence-empowered system design are triggering a research paradigm transformation: as THz research evolves from isolated component optimization toward system-level cross-disciplinary integration. This convergence paves the way for transformative applications spanning precision neurotherapeutics, ultrafast biosensing platforms, and cognitive radio networks.

This special issue, Emerging Terahertz Sciences and Technologies, captures this pivotal moment in the THz field, presenting a comprehensive exploration of THz advancements through seven seminal contributions—four comprehensive reviews and three original research articles. Collectively, these articles exemplify the synergistic interplay between foundational technological innovation and application-driven discovery, offering a roadmap from theoretical models to translational breakthroughs. The four review articles systematically review critical advancements in Coherent detection of pulsed terahertz waves, Terahertz channels in atmospheric conditions, beam-scanning antenna technologies, and Terahertz metamaterial biosensors, establishing a unified framework for cross-domain research. Complementing these, three research articles pioneer disruptive applications in neurotransmitter modulation, ultra-sensitive biosensing, and neuropsychiatric therapy, demonstrating how THz technologies can address grand challenges in high-sensitivity medical detection and high-precision neural modulation.

## 2 Coherent detection of pulsed terahertz waves

Coherent THz wave detection techniques employing solid-, gas-, and liquid-based methods each present unique advantages and limitations. Solid-state detectors offer high sensitivities and high signal-to-noise ratios but suffer from bandwidth limitations and low damage thresholds. Gas-phase methods enable long-distance detection with high damage tolerance, yet demand high-energy lasers due to low gas molecule density. Liquid-based approaches, while requiring minimal laser power and providing enhanced sensitivity through moderate molecular density, face constraints in bandwidth and signal quality due to optical dispersion and THz absorption [2]. These detection advancements have propelled THz spectroscopy and imaging into critical roles across material science, biomedicine, and security. Time-resolved THz spectroscopy unravels ultrafast semiconductor dynamics, including electron-phonon interactions, while THz spectral fingerprints enable label-free monitoring of protein conformational changes. Imaging applications span from tracking biomolecular activities to identifying explosives and pathogens. The emergence of intense THz sources has further amplified nonlinear material interactions, enabling unprecedented microscopic observations.

The dynamic properties of charge carriers in a substance determine the crucial electricity and heat characteristics for thermoelectric materials, but they are extremely difficult to diagnose for high-conductivity bulk materials due to Terahertz and other electromagnetic waves with very limited penetration depth. In a recent Science paper, Zhao and Chang et al. proposed a novel method by monitoring the ultra-fast dynamics of charge carrier performance in optical-pump reflection-style THz-probe spectroscopy measurements for high-conductivity bulk substance [3]. Based on the diagnosis, the slow decay constants of SnS_0.9_Se_0.1_ and SnS_0.9_Se_0.1_ + 0.03SnS_2_ are extracted as 300.3 ps and 195.2 ps, demonstrating the evidence of high carrier mobility even after introducing SnS_2_ in SnS_0.9_Se_0.1_. By conducting the THz static measurement under vacuum conditions, the reflection waves of SnS_0.9_Se_0.1_ + 0.03 SnS_2_ are higher than SnS_0.9_Se_0.1_ in both high-angle-incident and low-angle-incident modes, illustrating the improved carrier density. Overall, THz radiation with low energy (1 THz = 4.1 mV) provides a non-destructive solution to probe free carrier density and mobility via the photo-induced change in the surface conductivity, while being insensitive to the excitons and trapped carriers. THz technology can be anticipated to contribute to the semiconductor design and in situ characterization diagnosis, avoiding any artifacts due to the electronic contacts to the mesoporous material.

Undeniably, current challenges still exist: solids require bandwidth expansion and durability improvements, gases need energy-efficient excitation, and liquids demand sensitivity enhancements. Promising solutions include developing solids with higher electro-optic coefficients and damage resistance, optimizing liquids with reduced absorption and amplified nonlinearities, and exploring hybrid detection strategies. Future breakthroughs in these directions are poised to revolutionize THz applications, from fundamental studies of quantum materials to real-world deployments in medical diagnostics and threat detection. The continued refinement of these detection paradigms will ultimately dictate the scope and impact of THz technologies across scientific and industrial frontiers.

### 2.1 Reliable communication: from channel physics to adaptive antennas

The atmospheric channel fundamentally governs THz communication reliability, demanding physics-driven system designs. THz waves face severe molecular absorption, scattering by particles, and turbulence-induced scintillation effects [4]. Turbulence introduces rapid signal fluctuations, while urban multipath effects generate >50 ns delay spreads, degrading error-free transmission. Emerging terahertz communication systems require multidisciplinary innovations to overcome atmospheric challenges. Advanced AI-driven channel modeling must integrate multi-scale weather effects with real-time dynamics. Novel measurement methodologies should combine long-term outdoor campaigns, multi-frequency analysis, and specialized THz sounders. Weather-adaptive systems demand intelligent beamforming, cognitive radio architectures, and turbulence-resistant coding schemes. Security enhancements leverage quantum cryptography, atmospheric absorption-based secure zones, and AI-optimized interference patterns. Concurrently, material breakthroughs in high-power THz sources/sensors and reconfigurable intelligent surfaces promise hardware-level improvements. Dual-function systems exploiting THz’s environmental sensitivity could enable simultaneous communication and atmospheric monitoring. These coordinated advances across modeling, measurement, adaptation, and security domains will enable robust outdoor THz networks while informing next-generation wireless standards.

Terahertz beam-scanning technologies bridge historical microwave principles with THz-specific innovations, facing unique challenges due to submillimeter wavelengths. Four core methods dominate: mechanical motion (limited by speed/precision), phased arrays (burdened by insertion loss despite electronic/optical phase-shifting), frequency scanning via leaky-wave antennas/diffraction gratings (constrained by nonlinear beam distortion), and reconfigurable metasurfaces (enabling agile beam control via tunable materials like graphene/MEMS) [5]. Emerging solutions include topological waveguides for low-loss phased arrays and integrated microwave photonics for chip-scale THz systems. Metasurfaces evolve into intelligent variants with embedded signal detection and spatiotemporal modulation (STCMs), enabling unprecedented wave manipulation through space-time coding. Hu et al. [6] achieved ultrafast programmable temporal evolution through pixelated design using multi-materials and trigger switches. While photoconductive antennas leverage optical pumping for THz generation, persistent gaps remain in scalability and loss mitigation. Critical advances demand interdisciplinary synergy—combining Si/InP heterogeneous integration, high-speed switching mechanisms, and novel materials (GeTe, VO₂). Future priorities include stabilizing frequency-scanned beams, minimizing phased-array losses, and expanding STCM applications (e.g., real-time THz imaging). Building on microwave foundations while exploiting THz-material interactions, these innovations aim to mature THz systems for 6G communications and ultra-precision sensing.

### 2.2 Precision sensing: metamaterials redefine sensitivity limits

THz metamaterial biosensors are overcoming traditional sensitivity and specificity barriers through advanced resonance engineering and hybrid material integration, achieving atomic-level detection of biomarkers by amplifying frequency shifts and near-field interactions [7]. Surface functionalization with antibodies or aptamer-conjugated nanoparticles enables high-specificity targeting, while microfluidic-hydrogel systems mitigate water absorption interference, reducing THz signal attenuation in biofluids. Emerging multi-parameter platforms integrate machine learning to simultaneously analyze complex biomarkers in blood or urine. Innovations like phase-change material-embedded meta-surfaces and 3D-printed microfluidic-metamaterial hybrids promise tunable, real-time sensing for point-of-care diagnostics. Qiu et al. engineered two transmission spectra between picosecond time delays to eliminate typical measurement errors and achieved calibration-free detection [8]. And Qiu et al. proposed a novel strategy of using surface waves to achieve refractive sensing and fingerprint recognition, with a sensitivity of 215.5°/RIU, surpassing traditional meta-surface sensing methods [9]. By synergizing nanophotonic design, biochemical functionalization, and AI-driven analytics, THz metamaterial biosensors are redefining precision medicine, enabling early disease diagnosis at unprecedented sensitivity thresholds while addressing clinical practicality through robustness and scalability.

## 3 Exploration of biomedical applications

Due to the vibrational and rotational frequencies of biomolecules in the THz band, multiple studies have explored the effects of THz waves at specific frequencies on regulating ion channels, neurons, and animal behavior both theoretically and experimentally [[10], [11], [12], [13], [14], [15]]. In this special issue, three excellent works explore the biomedical applications of THz waves.

### 3.1 Retime-mapping terahertz vernier biosensor

Chang et al. introduced a THz vernier biosensor leveraging dual Mach-Zehnder interferometers in a three-channel metallic waveguide to exploit the optical vernier effect [16]. By superimposing slightly detuned THz interference spectra, the sensor achieves a sensitivity of 22.54 THz/RIU at 0.9 THz, with experimental areic mass sensitivity and accuracy of 10^7^ GHz/(g/mm²) and 10⁻⁸ g/mm²—surpassing single-interferometer performance by > 3,000%. Demonstrated through amino acid oxidation analysis, the label-free platform enables rapid identification of biochemical samples, showcasing its potential for ultra-sensitive THz sensing in wireless communications and high-precision diagnostics.

### 3.2 Neurotransmitter dedocking & photoneuromodulation

Zhu et al. introduce THz as a novel physical intervention for neurodegenerative diseases by disrupting catecholamine (CA) metabolite aggregation [17]. Electromagnetic stimulation at 44.5 THz dissociates dopamine (DA) ligands from dopamine receptor D2 (DRD2) binding sites via hydrogen bond cleavage, as validated by molecular docking. THz spectral analysis (0.5–50 THz) revealed CA-specific vibrational modes linked to conformational changes. The findings demonstrate THz radiation’s potential to mitigate neurotoxicity by clearing pathological CA accumulations, offering a non-invasive strategy for neural regulation and drug development in disorders like Parkinson’s and Alzheimer’s. Chang et al. demonstrated THz photoneuromodulation as a rapid, non-invasive therapy for depression, outperforming conventional SSRIs and PRXT [18]. THz irradiation at 34 THz stimulation increased serotonin (5-HT) levels by 107.5% within 2 min, suppressed overactive OFC glutamatergic neurons, and alleviated depression-like behaviors in mice for 1–2 weeks—far exceeding PRXT’s 30–120 min efficacy. Mechanisms involve frequency-specific molecular interactions and neural excitation-inhibition balance. While limited to male mice, the approach shows promise for clinical translation, addressing the delayed onset and side effects of current antidepressants. Future work will optimize parameters and explore sex-specific responses for broader neuropsychiatric applications.

## 4 Conclusion

THz technology is breaking through traditional application boundaries to become a revolutionary tool in communications, medical detection, and neuroscience. In communications, the co-optimization of dynamic beam-scanning antennas and atmospheric channel modeling will enable high-security, high-capacity integrated space-air-ground transmission. In biomedicine, the integration of metamaterial biosensors and THz-neuromodulation techniques pioneers a closed-loop precision medicine paradigm of “detection-mechanism-therapy”. This multiscale capability—from molecular vibration identification to neurotransmitter dynamics modulation—positions THz as the first universal technology bridging physics, engineering, and life science, offering unified solutions for healthcare, secure connectivity, and industrial innovation.

## CRediT authorship contribution statement

**Yuankun Sun:** Investigation, Methodology, Writing – original draft. **Shaomeng Wang:** Funding acquisition, Supervision, Writing – review & editing. **Yubin Gong:** Funding acquisition, Supervision, Visualization, Writing – review & editing.
